# Laser-tuned whispering gallery modes in a solid-core microstructured optical fibre integrated with magnetic fluids

**DOI:** 10.1038/srep17791

**Published:** 2015-12-03

**Authors:** Wei Lin, Hao Zhang, Bo Liu, Binbin Song, Yuetao Li, Chengkun Yang, Yange Liu

**Affiliations:** 1 Key Laboratory of Optical Information Science and Technology, Ministry of Education, Institute of Modern Optics, Nankai University, Tianjin 300071, China

## Abstract

A laser-assisted tuning method of whispering gallery modes (WGMs) in a cylindrical microresonator based on magnetic-fluids-infiltrated microstructured optical fibres (MFIMOFs, where MF and MOF respectively refer to magnetic fluid and microstructured optical fibre) is proposed, experimentally demonstrated and theoretically analysed in detail. The MFIMOF is prepared by infiltrating the air-hole array of the MOF using capillary action effect. A fibre-coupling system is set up for the proposed MFIMOF-based microresonator to acquire an extinction ratio up to 25 dB and a Q-factor as large as 4.0 × 10^4^. For the MF-infiltrated MOF, the light propagating in the fibre core region would rapidly spread out and would be absorbed by the MF-rod array cladding to induce significant thermal effect. This has been exploited to achieve a WGM resonance wavelength sensitivity of 0.034 nm/mW, which is ~20 times higher than it counterpart without MF infiltration. The wavelength response of the resonance dips exhibit linear power dependence, and owing to such desirable merits as ease of fabrication, high sensitivity and laser-assisted tunability, the proposed optical tuning approach of WGMs in the MFIMOF would find promising applications in the areas of optical filtering, sensing, and signal processing, as well as future all-optical networking systems.

In the past few decades, owing to their high Q-factor and small mode volume, whispering gallery mode (WGM) microresonators have been extensively investigated in various fundamental studies as well as engineering applications such as nonlinear optics^1^,^2^, cavity quantum electrodynamics^3^,^4^, bio-/chemical sensing^5^,^6^,^7^, optical signal processing^8^, and microcavity laser^9^,^10^. The WGM microresonators could be classified into versatile categories, including microdisk^11^, microring^12^, microsphere^13^, microbottle^14^ and capillary^15^, etc. Amongst these structures, due to their applicability for microfluidic applications and ease of being integrated with functional materials, open-cavity microresonators, such as microbottles and capillaries, have attracted growing research interests in developing photonic components with more intriguing functionalities for various applications, including fluidic bio-/chemical sensing^5^,^16^,^17^, localized laser excitation^18^,^19^, and field-dependent photonic devices^20^.

With the conceptual and fabrication technique progress of microstructured optical fibres (MOFs) since J. C. Knight and P. Russel’s pioneering studies in the 1990s^21^,^22^, MOF-based functional photonic devices have been extensively studied in the past few decades, such as fibre sensor^23^, dispersion management component^24^, nonlinear optical component^25^, tunable photonic device^26^ and microparticle trapper^27^. And moreover, by infiltrating functional materials into the periodic cross sectional air-hole array of the MOFs to control the eigenmode propagation characteristics along the fibre axis, it would be convenient to equip the MOFs with a good variety of functionalities. More importantly, the presence of circularly symmetric cross sectional microstructures in MOFs provides a possibility of developing MOF-based WGM microresonators with controllable optical properties based on the infiltration of functional materials.

In this paper, we have presented a WGM microresonator based on a magnetic fluid (MF) infiltrated MOF (MFIMOF). The fibre-coupled WGM spectra of the microresonators with different diameters show high extinction ratios (ERs) at eigenmode resonance wavelengths. Since the refractive index of the deep coloured MFs is close to the silica background, the MOF-based microresonator could be regarded as a cylindrical cavity resonator comprising strongly thermal absorption medium. Based on this simplification, we calculate the orders of the WGM eigenmodes supported by the proposed MOF-based microresonator, which is in good agreement with our experimental observation. The fibre cross sectional electric field distributions are also numerically simulated and theoretically analysed for the experimentally observed WGM resonance dips. Furthermore, we theoretically analyse and experimentally investigate laser-assisted tunability of the WGMs in consideration of the photo-thermal effect of the MFs. For comparison purpose, we also investigate the contribution of the MFs to WGM tunability by employing a MOF-based microresonator without infiltrating the MFs. Our proposed MOF-based microresonator possesses several desirable features such as highly sensitive laser-assisted tunability, high linear power dependence and ease of fabrication, which make it a good candidate for potential applications in the areas of optical filtering, sensing, and signal processing as well as future all-optical networking systems.

## Results and Discussion

### Observation of the WGMs in MFIMOF

**Figure 1:**
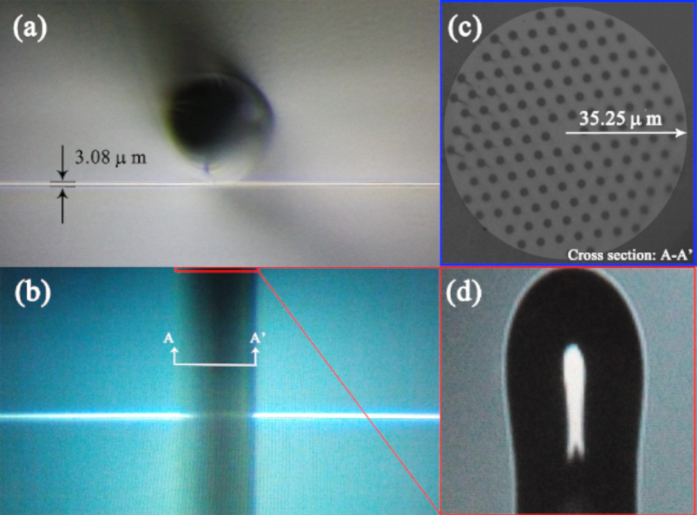
Photographs of the MFIMOF-based WGM microresonator. (**a**) and (**b**) respectively correspond to the front and top views of the microresonator in contact with the microfibre; (**c**) cross sectional image of the experimentally used MOF; the air-hole array cladding has an equivalent radius of about 35.25 μm; (**d**) micrograph of the fused free facet of the MFIMOF.

In the work presented in this paper, the fibre-taper-based WGM excitation technique firstly reported by J. C. Knight *et al.* is employed to investigate the WGM properties of the proposed MFIMOF^28^. As the input light enters the fibre taper, the evanescent field would be excited. Owing to the mode overlapping between the taper evanescent field and the WGMs in the MFIMOF, part of the evanescent field would be coupled into the WGMs propagating inside the microresonator around the contact region. Therefore, WGM resonance dips could be acquired in the transmission spectrum of the fibre taper. The fibre-coupled system for the proposed MFIMOF was proposed and constructed as described in Methods section. Figure 1(a,b) show the front and top views of the microresonator in contact with the microfibre, respectively. The cross sectional image of the MOF is given in Fig. 1(c), from which it could be seen that the air-hole array cladding radius is about 35.25 μm. It should be mentioned that the free facet of the MFIMOF should be fused in order to prevent the MFs from leaking out when pump light is injected into the MOF, as shown in Fig. 1(d).

**Figure 2:**
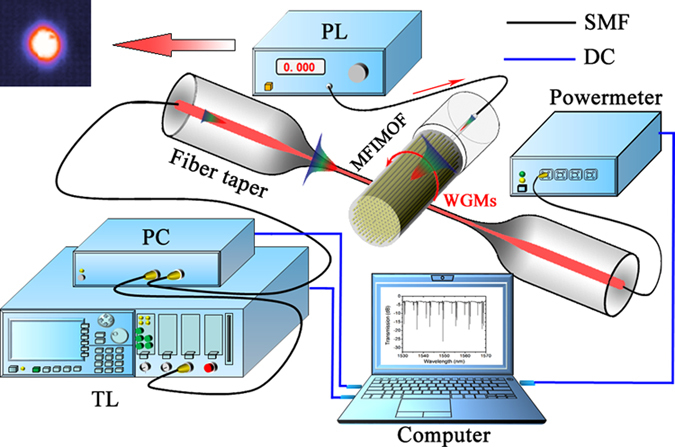
Schematic diagram of the performance test system of the proposed MFIMOF-based microresonator. TL: tunable laser, PC: polarization controller, PL: pump laser, SMF: single-mode fibre, DC: data cable, MFIMOF: magnetic-fluid-infiltrated microstructured optical fibre. Inset shows the mode field profile of the pump laser. Figure 2 was created by using Microsoft Office.

A performance test system is utilized to investigate the optical tunability of the WGMs excited in the MFIMOF-based microresonator, as shown in Fig. 2. The optical spectrum of the microresonator is measured by an insertion loss test system (produced by Agilent, USA), which consists of a tunable laser (TL, operation wavelength ranges from 1530 nm to 1570 nm with a wavelength step of 3 pm), a polarization controller (PC) and a powermeter. The inset of Fig. 2 gives the mode field profile of the pump laser. All of these devices are connected with a computer control platform using data cable (DC). When the WGM fibre coupling system is placed between the PC and the powermeter through SMFs, the transmission spectrum of the microfibre could be measured and acquired by the computer. As the input pump laser power changes, the optical tunability of the WGMs could be investigated by analyzing the transmission spectral characteristics of the microfibre carrying the information on the WGMs in the MFIMOF.

**Figure 3:**
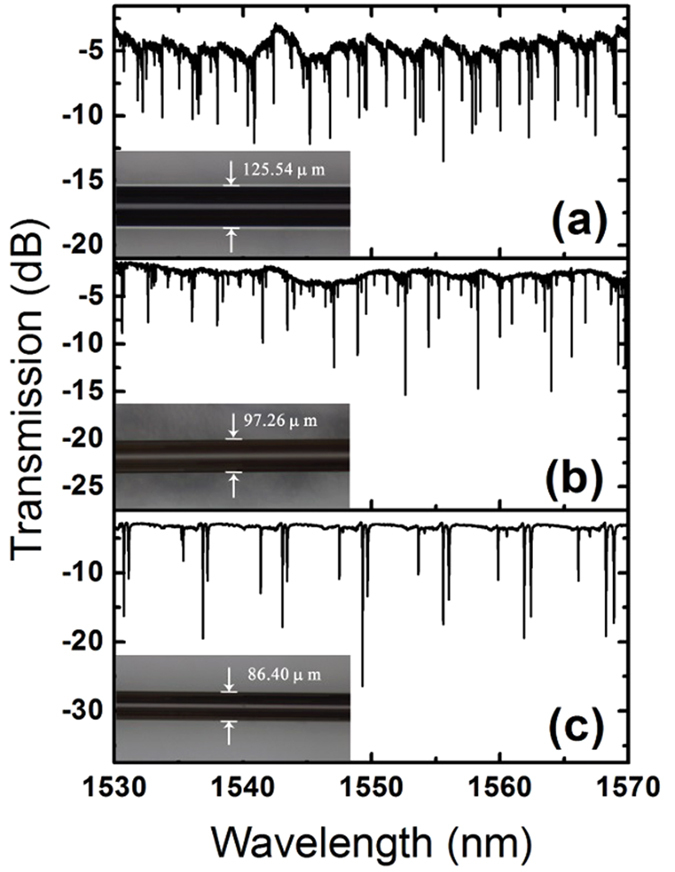
Transmission spectra of the microfibre in the presence of the MFIMOF-based microresonator with respective diameters of (a) 125.54 μm (b) 97.26 μm (c) 86.4 μm.

To improve spectral tuning quality of the proposed MOF-based microresonator, the WGMs excited in the MFIMOF are further purified by reducing the diameter of the MFMOF through HF acid etching procedure. Figure 3 illustrates the output spectra of the microfibre in the presence of the microresonators with respective diameters of 125.54 μm, 97.26 μm and 86.4 μm. It is apparent that only three types of WGMs could be excited when the diameter decreases to 86.4 μm.

### Theoretical analysis on WGMs excited in the MFIMOF

**Figure 4:**
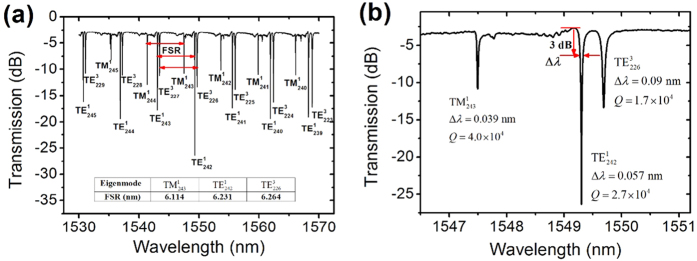
Transmission spectrum of the microfibre in presence of the MFIMOF microresonator with a diameter of 86.4 μm. (**a**) Mode orders corresponding to different resonance dips. The FSRs for 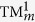, 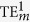 and 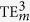 modes are also calculated (**b**) Enlarged transmission spectrum around 1550 nm. The FWHM and Q factors for 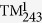, 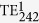 and 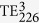 modes are also calculated.

Due to its better WGM purity, the MFIMOF with a diameter of 86.4 μm is selected to investigate the WGM properties of the MFIMOF. The azimuthal and radial quantization numbers: m and l of the WGMs in the proposed MFIMOF is calculated by solving the eigen equation (7) in Methods section. Figure 4 shows transmission spectrum of the microfibre for the MFIMOF with a diameter of 86.4 μm. It could be seen that the excited WGMs belong to 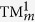, 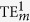 and 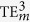 modes classifications, and the free spectral ranges (FSRs) increases with the increment of WGM resonance wavelength. The experimentally measured typical FSRs for 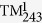, 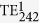 and 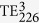 modes are 6.114 nm, 6.231 nm 6.264 nm, respectively. The ERs of the resonance dips reach 23 dB, which is acceptable for most optical sensing as well as optical communications applications. An enlarged transmission spectrum around 1550 nm is given in Fig. 4(b). The full widths at half maximum (FWHMs) of the resonance dips for 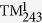, 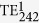 and 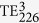 modes are measured and their Q factors are also calculated using Q = *λ*/Δ*λ*^29^. For the first-order resonance dips, the FWHMs turn out 0.039 nm and 0.057 nm and their Q factors reach 4.0 × 10^4^ and 2.7 × 10^4^ for TM and TE modes, respectively. And for the third-order WGM, the FWHM is 0.09 nm and its Q factor is 1.7 × 10^4^.

**Figure 5:**
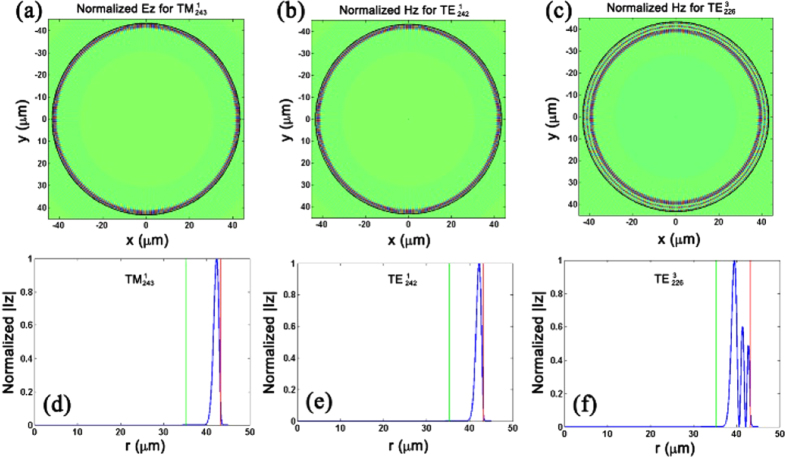
Mode profiles and the radius-dependent distributions of Iz. (**a**) Normalized Ez distribution for 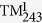 mode; (**b**,**c**) correspond to the normalized Hz for 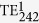 and 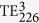 modes. The dark circles in the mode profiles refer to the interface between the MOF and air; (**d**–**f**) respectively correspond to the radius-dependent normalized intensity distributions for these three WGMs. The red line refers to the interface between the MOF and air while the green line represents the equivalent interface between the outer silica cladding and the MF-rod-array cladding.

Figure 5 illustrates the mode profiles for 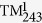, 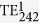 and 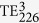 modes, respectively. Figure 5(a) is the z-axis component of the normalized electric field *E*_z_ for 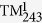 mode. Figure 5(b,c) correspond to the z-axis components of the normalized magnetic field *H*_*z*_ for 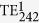 and 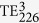 modes respectively. The related radius-dependent distributions for the z-axis component of normalized intensity *I*_*z*_ are also illustrated in Fig. 5. The red line refers to the interface between the MOF and air while the green line represents the equivalent interface between the outer cladding and the MF-rod-array cladding. Simulation results indicate that light would propagate along the inner wall of the MOF for the first-order WGMs. With the increment of radial quantization number *l*, most WGM energy would gradually disperse inward. When the WGMs disperse into the inner MF-rod-array cladding, due to the strong absorption and scattering effect introduced by the MF-rod-array, these modes would experience high transmission loss and would no longer be supported in the MFIMOF microresonator. Hence, as the thickness of the outer silica cladding decreases, the order of supported WGMs in the MFIMOF resonator would reduce accordingly. This is basic principle for WGM purification through chemical etching procedure.

### Theoretical analysis on WGM Tuning properties

When a continuous light wave is injected into the fibre taper, the light would be coupled into the MFIMOF resonator through the interaction between the evanescent field over the microfibre and WGMs inside the microcavity. When stable optical oscillation is established, the transmittance of the microresonator could be described as equations (1) and (2) 30. It should be noted that the coupling between these three WGMs could be actually neglected due to the phase mismatching between them.


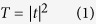



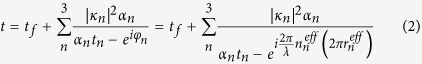


where *t* is the amplitude transmittance; *t*_*f*_ and *t*_*n*_refer to the self-coupling coefficients of the fibre mode and the WGMs, respectively; subscript *n* respectively correspond to 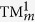, 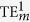 and 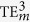 modes; *κ*_*n*_ is the coupling coefficient between the fibre mode and WGMs; *α*_*n*_ is the transmittance of the WGMs propagating in the microresonator; *φ*_*n*_ represents the phase variation of the WGMs propagating after for one cycle; *n*_*n*_^*eff*^ and *r*_*n*_^*eff*^ refer to the effective refractive index and radius of the WGMs, respectively, which co-determines the WGM resonance wavelength through 
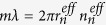
. When the pump laser is injected into the MFIMOF, light would disperse into the cladding region and would be rapidly absorbed by the MF rods^31^,^32^. Thus the MFIMOF will be heated and consequently *n*_*n*_^*eff*^ and *r*_*n*_^*eff*^ would vary due to the thermo-optic and thermal expansion effect, which could be expressed as:


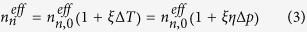



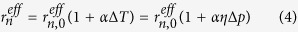


where *ξ* and *α* refer to thermo-optic and thermal expansion coefficients, respectively, and their values are 8.3 × 10^−6^ and 0.55 × 10^−6^ for silica material; 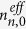 and 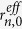 are respectively defined as initial effective refractive index and effective radius before the pump laser is injected into the MFIMOF; *η* refers to the correlation coefficient between temperature and pump laser power, which depends on the absorption coefficient and thermal conductivity of the MFs as well as geometry of the MOF^20^,^33^. If the wavelength dependences of 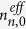 and 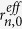 are taken into account, the power-tuning sensitivity of particular WGM could be expressed as:


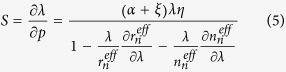


**Figure 6:**
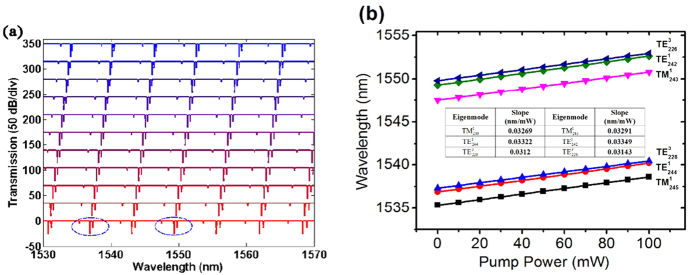
(**a**) Simulated transmission spectral evolution for different pump powers with an offset of 50 dB/div according to Eqs (3)~(6) by using the parameters given in Table 1 with a laser power step of 10 mW. (**b**) Simulated resonance wavelength as functions of pump laser power for the six experimentally selected WGM resonance dips.

According to equations (1)~(4), the transmission spectra of the microfibre under different pump powers are simulated, as shown in Fig. 6(a). The laser power step is set to 10 mW in the simulation process. The parameters used in our simulation are summarized in the following Table 1.

It could be seen that as the applied pump laser power increases, the WGM resonance dips move toward longer wavelength region. Figure 6(b) shows WGM resonance wavelength as functions of applied pump laser power for the six selected WGM resonance dips. It is apparent that the resonance wavelength linearly increases with the increment of pump laser power, and the wavelength sensitivities respectively reach 0.03269 nm/mW, 0.03322 nm/mW, 0.0312 nm/mW, 0.03291 nm/mW, 0.03349 nm/mW and 0.03141 nm/mW for 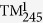, 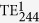, 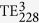, 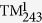, 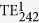, and 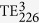 modes. Also, we could find that the resonance dips with longer wavelength possess higher wavelength sensitivity for the same WGM order, which is in accordance with equation (5). In addition, according to our simulation results in the above table, 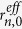 of the WGMs with *l* = 3 possesses stronger wavelength dependence. Therefore, the resonance dips for the third order WGMs would exhibit lower wavelength sensitivity to the applied pump laser power according to equation (5).

### Experimental results and discussion on WGM Tuning properties

**Figure 7:**
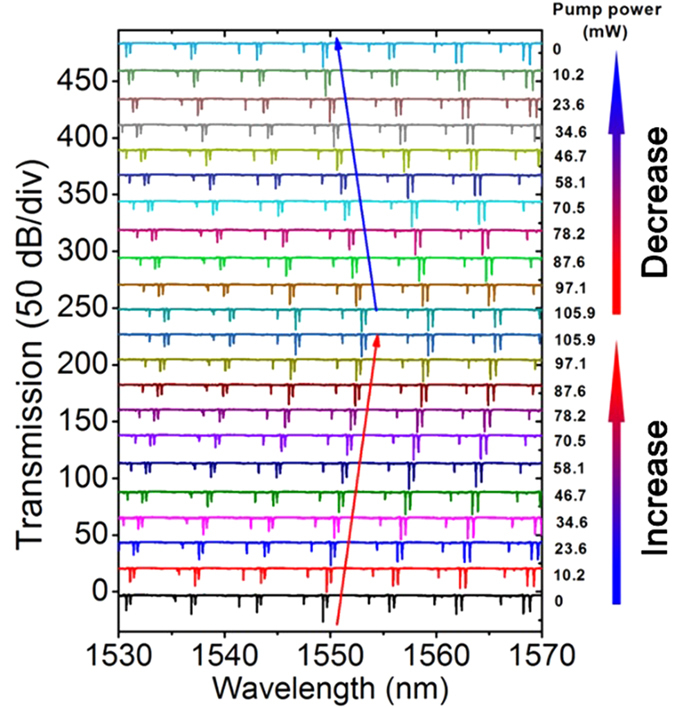
Transmission spectral evolution of the microfibre with an offset of 50 dB/div for pump power increase and decrease cases, respectively. The arrows indicate the resonance wavelength shift direction.

We have experimentally investigated the tuning properties of the MFIMOF-based microresonator by employing the performance test system schematically illustrated in Fig. 2. Figure 7 shows the transmission spectral evolutions of the MFIMOF-based microresonator for the pump power increase and decrease cases, respectively. As the laser power gradually increases from 0 mW to 105.9 mW, the resonance dips exhibit linear red shift, which is in good agreement with the above theoretical analysis. However, as the laser power is turned down, the resonance dips would show linear blue shift. It should be noted that the resonance dips would generally return to their initial wavelength positions after a full measurement cycle, which indicates the good spectral reversibility of our proposed WGM tuning approach.

**Figure 8:**
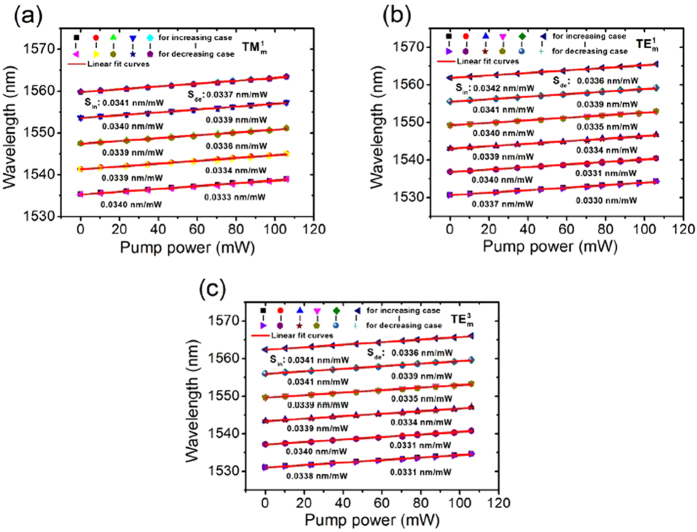
WGM resonance wavelength as functions of applied pump laser power. (**a**–**c**) respectively correspond to the resonance wavelength responses of 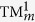, 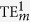 and 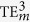 modes within a full measurement cycle, respectively. The dot-pairs refer to the experimental data under the same pump power.

Figure 8 shows pump power dependences of the WGM resonance wavelength for our proposed MFIMOF-based microresonator. Figure 8(a–c) correspond to the resonance wavelength as functions of pump power within a full measurement cycle for 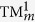, 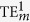 and 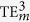 modes, respectively. The dot-pairs in these three figures refer to the experimental data under the same pump laser power. It is clear that all of the resonance dips move toward longer wavelength region with highly linear sensitivities to the applied pump power. Linear fitting of the wavelength response curves indicate that the linear coefficients are larger than 0.99 for all of the resonance dips. It could be also seen that the tuning sensitivities of these WGMs reach about 0.034 nm/mW. Additionally, the resonance dips with longer wavelength exhibit larger sensitivity to the pump power, which is in agreement with the above theoretical analysis. However, the WGMs with different radial quantization numbers exhibit close wavelength sensitivities, which could be attributed to the compensation effect of the correlation coefficient *η* for different WGMs. The laser-induced thermal effect resulting from the absorption of the MF rod-array cladding would actually lead to uneven cross sectional temperature distribution, as reported in Mazumder *et al.*’s work^34^. Therefore, temperature would be higher for those regions closer to the central cross sectional area, which results in a center-concentrated correlation coefficient profile over the fibre cross section. Since the effective radius 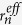 of 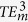 mode is smaller than those of 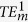 and 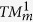 modes, the correlation coefficient of 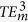 mode would be larger than those of 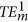 and 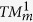 modes, which causes the enhancement of wavelength tuning sensitivity. The uneven cross sectional temperature distribution would have an impact on the wavelength tuning sensitivity in opposition to the wavelength dependences of effective radius and effective refractive indices. Hence, in consideration of these factors, the third-order WGMs would actually exhibit wavelength sensitivity close to those of 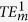 and 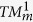 modes.

**Figure 9:**
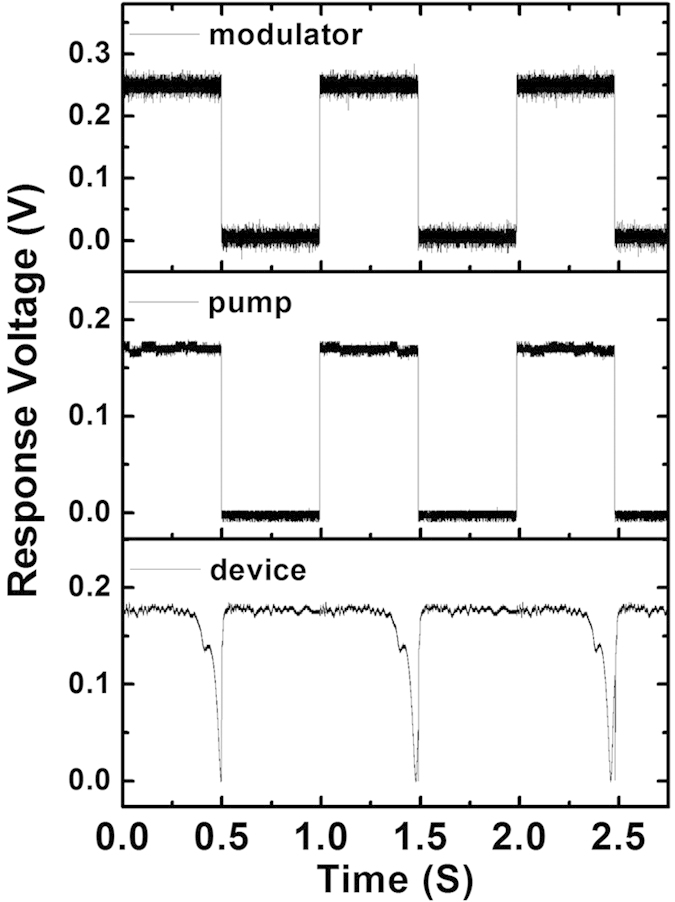
(**a**) Input signal of the modulator; temporal response of (**b**) the pump laser (**c**) the proposed MFIMOF.

In order to investigate the dynamic response of the WGM tunability, the pump light is modulated by using a driving current modulator. The input signal of the modulator has a duty cycle of 50% and frequency of 1 Hz. Figure 9 shows the input signal of the modulator and the temporal transmittance responses of the pump laser as well as the proposed device. As shown in this figure, the periodic optical transmittance indicates that the tunability of the WGM in MFIMOF has good stability and repeatability. The rising and dropping time of our proposed device are 62.8 ms and 448 ms, respectively, which generally agree with Liu *et al.*’s work^20^. Thus, our proposed MFIMOF would be more suitable for low speed optical signal modulation applications.

**Figure 10:**
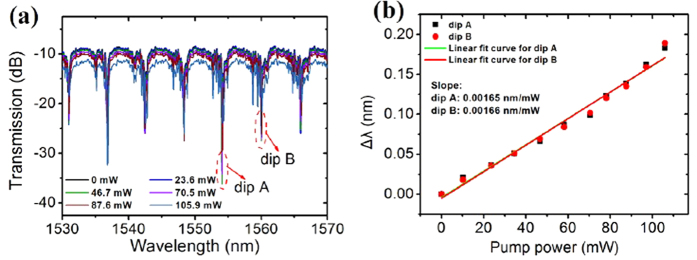
(**a**) Transmission spectral evolution of the microfibre without MF infiltration for different applied pump powers (**b**) Resonance wavelength shift as functions of pump laser power for dip A and dip B, respectively.

To investigate the contribution of MF infiltration to the WGM excitation as well as resonance wavelength tuning properties, we have also conducted a laser-assisted WGM tuning experiment for the MOF-based microresonator without MF infiltration. Figure 10(a) shows the transmission spectral evolution of the microfibre for different pump laser powers. It could be seen that the resonance dips show rather slight wavelength shift with the increment of pump laser power. Figure 10(b) gives the wavelength shift as functions of the pump power for the two selected resonance dips, which show highly linear laser power dependence with respective wavelength sensitivities of 0.00165 nm/mW and 0.00166 nm/mW for dip A and dip B. In comparison with the experimental results of the MFIMOF, the wavelength tunability has been significantly improved by enhancing the laser-induced thermal effect using magnetic nanoparticle fluids. The wavelength sensitivity of the MFIMOF is about twenty times as the one without MF infiltration, which could be attributed to the MF-induced large correlation coefficient. Therefore, it would be simple but effective to further improve the WGM tuning sensitivity by using more appropriate materials with higher absorption coefficient and thermal conductivity.

## Conclusions

We have presented and experimentally demonstrated a laser-assisted WGM tuning approach based on a MOF-based microresonator integrated with magnetic fluid nanoparticles. For the fibre-coupled MFIMOF-based microresonator, a high ER up to 23 dB and a Q-factor up to 4.0 × 10^4^ have been experimentally achieved. Due to the high absorption coefficient and thermal conductivity of the MFs, the photo-thermal tunability of the WGMs in MOFs could be significantly enhanced. The WGM resonance wavelength tuning sensitivity reaches 0.034 nm/mW, which is about twenty times as that of the MOF-based microresonator without MF infiltration. The laser power dependence of resonance wavelength shows high linearity for an applied pump laser power range of 0 mW to 105.9 mW. Our theoretical simulation on WGM tuning properties for the proposed WGM microresonator based on a photo-thermal effect model is in agreement with our experimental measurement results, and the theoretical analysis also indicates that by employing more appropriate materials with stronger thermal absorption and higher thermal conductivity, the WGM tuning sensitivity could be further improved. The proposed MFIMOF-based microresonator possesses several desirable merits such as linear laser power dependence, ease of fabrication, and laser-assisted noncontact manipulation, showing great promise for potential applications in all-optical fibre communications, fibre-optic sensing and future all-optical networking systems. And moreover, the cross sectional microstructures of MOFs provides more degrees of freedom for developing novel WGM-based functional photonic devices.

## Methods

### Construction of the performance test system

The experimentally used solid-core MOF (LMA-8 manufactured by NKT Photonics, Denmark), possesses a hexagonal air-hole array cladding with a duty cycle of 0.42 and an average air-hole diameter of 2.32 μm. The dioctyl-sebacate-based magnetic nanoparticle fluids (Hinano-FF5 produced by Hinano, Japan) are infiltrated into the cladding air-holes of the MOF based on capillary action effect. Due to the presence of circularly symmetric microstructures over the fibre cross section, the MFIMOF could act as a WGM resonator with highly thermal absorption medium inside. The MFIMOF is fixed onto a fibre holder on a micro-actuated platform and spliced with a 980 nm pump laser (PL) using a segment of standard single-mode fibre (SMF). In order to effectively excite WGMs in the MFIMOF, a microfibre with a diameter of 3.08 μm is fabricated by using a fibre tapering machine (produced by E-Otron, China). A section of standard SMF is heated by hydrogen flame and stretched till the tapered segment length is about 29 mm. The flame is about 5 mm in width and stands still during the whole tapering process. Then the tapered fibre is straightly tightened and adhered on a U-shape frame fixed on the rotation platform. By moving the MFIMOF toward the microfibre till close contact while keeping the perpendicularity between them, an effective WGM fibre coupling system could be set up.

### Eigenequation for WGMs in the MFIMOF

For the WGMs propagating in the MFIMOF, the time-independent axial field distribution could be described by *Ψz*(*r*)exp(*imϕ*), where exp(*imϕ*) is the azimuthal-dependent phase term and m is the azimuthal quantization number. *Ψz*(*r*) is the radial-dependent magnetic mode component for TE mode or electric mode field for TM mode in z-axial component. Since the equivalent RI of the hole-array cladding is close to the silica background, the MFIMOF-based microresonator could be simplified as a cylindrical resonator. Thus, *Ψz*(*r*) could be expressed as^35^:


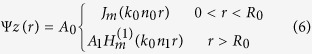


where *R*_0_ is the radius of the selected MFIMOF, which is equal to 43.2 μm; *n*_0_ and *n*_1_ represent the refractive indices of the quartz background of the MFIMOF and air, respectively; *k*_0_ is the wavenumber in vacuum; *A*_0_ and *A*_1_ are constants. According to the boundary conditions, the eigenequation of the MFIMOF-based microresonator could be written as:


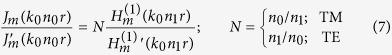


By solving the above eigenequation, the resonance wavelengths of the WGMs could be calculated and the order of the WGMs, including the azimuthal and radial quantization numbers: *m* and *l*, could be determined.

## Additional Information

**How to cite this article**: Lin, W. *et al.* Laser-tuned whispering gallery modes in a solid-core microstructured optical fibre integrated with magnetic fluids. *Sci. Rep.* **5**, 17791; doi: 10.1038/srep17791 (2015).

## Figures and Tables

**Table 1:** Parameters used in the simulation process.

	*t*_*f*_ a	*η*	*κ*_*n*_	*t*_*n*_ b	*α*_*n*_	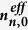 c	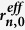 d
TE_m_^1^	0.9946	2.5	0.075	0.9972	0.995	1.46274–1.24735 * 10^−5^ * *λ*	41.7736–2.79607 * 10^−4^ * *λ*
TE_m_^3^			0.06	0.9992		1.46335–1.28406 * 10^−5^ * *λ*	41.59453–0.00192 * *λ*
TM_m_^1^			0.04	0.9982		1.46287–1.36518 * 10^−5^ * *λ*	42.54287–6.66597 * 10^−4^ * *λ*

^a^*t*_*f*_ is calculated using 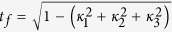.

^b^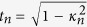.

^c^
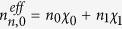
, where 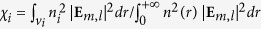 (*i* = 0,1). 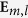 is the electric field that could be calculated according to the above WGM analysis method. In addition, the wavelength-dependent dispersion could be taken into account by performing linear fitting to the calculated 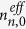 as a function of resonance wavelength. The coefficient of determination is 1. The unit of *λ* is nm.

^d^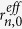 can be calculated using the 
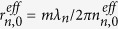
 and linear fitting of 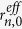 as a function of resonance wavelength shows a coefficient of determination of 1.

## References

[b1] Pfeifle J. *et al.* Optimally Coherent Kerr Combs Generated with Crystalline Whispering Gallery Mode Resonators for Ultrahigh Capacity Fiber Communications. Phys. Rev. Lett. 114, 093902 (2015). 25793816 10.1103/PhysRevLett.114.093902

[b2] Vukovic N. *et al.* Ultrafast optical control using the Kerr nonlinearity in hydrogenated amorphous silicon microcylindrical resonators. Sci. Rep. 3, 2885 (2015). 24097126 10.1038/srep02885 PMC3791441

[b3] Park Y., Cook A. K. & Wang H. Cavity QED with Diamond Nanocrystals and Silica Microspheres. Nano Lett. 6, 2075–2079 (2006). 16968028 10.1021/nl061342r

[b4] Vahala K. J. Optical microcavities. Nature 424, 839–846 (2003). 12917698 10.1038/nature01939

[b5] Zhu H. *et al.* X. Integrated Refractive Index Optical Ring Resonator Detector for Capillary Electrophoresis. Anal. Chem. 79, 930–937 (2007). 17263318 10.1021/ac061279q

[b6] Vollmer F. & Arnold S. Whispering-gallery-mode biosensing: label-free detection down to single molecules. Nat. Methods 5, 591–596 (2008). 18587317 10.1038/nmeth.1221

[b7] Armani A. M. *et al.* Label-Free, Single-Molecule Detection with Optical Microcavities. Science 317, 783–787 (2007). 17615303 10.1126/science.1145002

[b8] Wang P. *et al.* Packaged, high-Q, microsphere-resonator based add–drop filter. Opt. Lett. 39, 5208–5211 (2014). 25166111 10.1364/OL.39.005208

[b9] He L., Özdemir Ş. K. & Yang L. Whispering gallery microcavity lasers. Laser Photonics Rev. 7, 60–82 (2013)

[b10] Franois A., Rowland K. J. & Monro T. M. Highly efficient excitation and detection of whispering gallery modes in a dye-doped microsphere using a microstructured optical fiber. Appl. Phys. Lett. 99, 141111 (2011).

[b11] Liu Y., Marcelo D., Vladimir A. & Kartik S. Electromagnetically Induced Transparency and Wideband Wavelength Conversion in Silicon Nitride Microdisk Optomechanical Resonators. Phys. Rev. Lett. 110, 223603 (2013). 23767723 10.1103/PhysRevLett.110.223603

[b12] Cai D. *et al.* High Q-factor microring resonator wrapped by the curved waveguide. Sci. Rep. 5, 10078 (2015). 25993265 10.1038/srep10078 PMC4438724

[b13] Spillane S. M., Kippenberg T. J. & Vahala K. J. Ultralow-threshold Raman laser using a spherical dielectric microcavity. Nature 415, 621–623 (2002). 11832940 10.1038/415621a

[b14] Pöllinger M., O’Shea D., Warken F. & Rauschenbeutel A. Ultrahigh-Q Tunable Whispering-Gallery-Mode Microresonator. Phys. Rev. Lett. 103, 053901 (2009). 19792499 10.1103/PhysRevLett.103.053901

[b15] Shopova S. I. *et al.* On-Column Micro Gas Chromatography Detection with Capillary-Based Optical Ring Resonators. Anal. Chem. 80, 2232–2238 (2008). 18271605 10.1021/ac702389x

[b16] Li M. *et al.* Self-Referencing Optofluidic Ring Resonator Sensor for Highly Sensitive Biomolecular Detection. Anal. Chem. 85, 9328−9332 (2013). 23992426 10.1021/ac402174x

[b17] Scholten K., Fan X. & Zellers E. T. A microfabricated optofluidic ring resonator for sensitive, high-speed detection of volatile organic compounds. Lab Chip 14, 3873–3880 (2014). 25131718 10.1039/c4lc00739e

[b18] Aubry G. *et al.* A multicolor microfluidic droplet dye laser with single mode emission. Appl. Phys. Lett. 98, 111111 (2011).

[b19] Lee W., Luo Y., Zhu Q. & Fan X. Versatile optofluidic ring resonator lasers based on microdroplets. Opt. Express 19, 19668–19674 (2011). 21996908 10.1364/OE.19.019668

[b20] Liu Y. *et al.* All-optical tuning of a magnetic-fluid-filled optofluidic ring resonator. Lab Chip 14, 3004–3010 (2014). 24941312 10.1039/c4lc00236a

[b21] Knight J. C. Photonic crystal fibres. Nature 424, 847–851 (2003). 12917699 10.1038/nature01940

[b22] Russel P. Photonic crystal fibers. Science 299, 358–382 (2003). 12532007 10.1126/science.1079280

[b23] Zhang Y. *et al.* Liquid core photonic crystal fiber sensor based on surface enhanced Raman scattering. Appl. Phys. Lett. 90, 193504 (2007).

[b24] Renversez G., Kuhlmey B. & McPhedran R. Dispersion management with microstructured optical fibers: ultraflattened chromatic dispersion with low losses. Opt. Lett. 28, 989–991 (2003). 12836755 10.1364/ol.28.000989

[b25] Biancalana F. *et al.* Emergence of Geometrical Optical Nonlinearities in Photonic Crystal Fiber Nanowires. Phys. Rev. Lett. 105, 093904 (2010). 20868164 10.1103/PhysRevLett.105.093904

[b26] Du J. *et al.* Electrically tunable Sagnac filter based on a photonic bandgap fiber with liquid crystal infused. Opt. Lett. 33, 2215–2217 (2008). 18830356 10.1364/ol.33.002215

[b27] Schmidt O. A. *et al.* Reconfigurable optothermal microparticle trap in air-filled hollow-core photonic crystal fiber. Phys. Rev. Lett. 109, 024502 (2012). 23030165 10.1103/PhysRevLett.109.024502

[b28] Knight J. C. *et al.* Phase-matched excitation of whispering-gallery-mode resonances by a fiber taper. Opt. Lett. 22, 1129–1131 (1997). 18185771 10.1364/ol.22.001129

[b29] Reynolds T. *et al.* Optimization of whispering gallery resonator design for biosensing applications. Opt. Express 23, 17067–17076 (2015). 26191715 10.1364/OE.23.017067

[b30] Zhang X. *et al.* Ultraprecise Resonance Wavelength Determination for Optofluidic Sensing Applications. IEEE Photon. Technol. Lett. 27, 399–402 (2015).

[b31] Gao R., Jiang Y. & Abdelaziz S. All-fiber magnetic field sensors based on magnetic fluid-filled photonic crystal fibers. Opt. Lett. 38, 1539–1541 (2013). 23632544 10.1364/OL.38.001539

[b32] Candiani A. *et al.* A loss-based, magnetic field sensor implemented in a ferrofluid infiltrated microstructured polymer optical fiber. Appl. Phys. Lett. 104, 111106 (2014).

[b33] Zhao P. *et al.* Iron-oxide nanoparticles embedded silica microsphere resonator exhibiting broadband all-optical wavelength tunability. Opt. Lett. 39, 3845–3848 (2014). 24978752 10.1364/OL.39.003845

[b34] Mazumder J. & Steen W. M. Heat transfer model for CW laser material processing. J. Appl. Phys. 51, 941–947 (1980).

[b35] Mie G. Beitrage zur optic triiber medien, speziell kolloidaler metallosungen. Ann. Phys. 330, 377–445 (1908).

